# Shoulder muscle endurance: the development of a standardized and reliable protocol

**DOI:** 10.1186/1758-2555-3-1

**Published:** 2011-01-11

**Authors:** Jean-Sébastien Roy, Bryan Ma, Joy C MacDermid, Linda J Woodhouse

**Affiliations:** 1 Centre for Interdisciplinary Research in Rehabilitation and Social Integration, Quebec City, Quebec, Canada; 2 Department of Rehabilitation, Faculty of Medicine, Laval University, Quebec City, Quebec, Canada; 3 School of Rehabilitation Science, McMaster University, Hamilton, Ontario, Canada; 4 Hand and Upper Limb Centre, St. Joseph's Health Centre, London, Ontario, Canada; 5 Department of Surgery, Holland Orthopaedic & Arthritic Hospital of Sunnybrook Health Sciences Centre, Toronto, Ontario, Canada; 6 Departments of Rehabilitation and Orthopaedics, Hamilton Health Sciences, Hamilton, Ontario, Canada

## Abstract

**Background:**

Shoulder muscle fatigue has been proposed as a possible link to explain the association between repetitive arm use and the development of rotator cuff disorders. To our knowledge, no standardized clinical endurance protocol has been developed to evaluate the effects of muscle fatigue on shoulder function. Such a test could improve clinical examination of individuals with shoulder disorders. Therefore, the purpose of this study was to establish a reliable protocol for objective assessment of shoulder muscle endurance.

**Methods:**

An endurance protocol was developed on a stationary dynamometer (Biodex System 3). The endurance protocol was performed in isotonic mode with the resistance set at 50% of each subject's peak torque as measured for shoulder external (ER) and internal rotation (IR). Each subject performed 60 continuous repetitions of IR/ER rotation. The endurance protocol was performed by 36 healthy individuals on two separate occasions at least two days apart. Maximal isometric shoulder strength tests were performed before and after the fatigue protocol to evaluate the effects of the endurance protocol and its reliability. Paired *t*-tests were used to evaluate the reduction in shoulder strength due to the protocol, while intraclass correlation coefficients (ICC) and minimal detectable change (MDC) were used to evaluate its reliability.

**Results:**

Maximal isometric strength was significantly decreased after the endurance protocol (*P *< 0.001). The total work performed during the last third of the protocol was significantly less than the first third of the protocol (P < 0.05). The test-retest reliability of the post-fatigue strength measures was excellent (ICC >0.84).

**Conclusions:**

Changes in muscular performance observed during and after the muscular endurance protocol suggests that the protocol did result in muscular fatigue. Furthermore, this study established that the resultant effects of fatigue of the proposed isotonic protocol were reproducible over time. The protocol was performed without difficulty by all volunteers and took less than 10 minutes to perform, suggesting that it might be feasible for clinical practice. This protocol could be used to induce local muscular fatigue in order to evaluate the effects of fatigue on shoulder kinematics or to evaluate changes in shoulder muscle endurance following rehabilitation.

## Background

Several risk factors have been identified for the development of rotator cuff disorders, including repetitive use of the shoulder [1]. Repetitive arm movements are a major component of several workplace tasks as well as many sporting and leisure activities [2,3]. Shoulder muscle fatigue has been proposed as a possible link to explain the association between repetitive arm use and the development of rotator cuff disorders. The hypothesis being that a series of continuous repetitive muscle contractions will inevitably lead to reduced capability in the prime mover [3,4] resulting in altered motor recruitment patterns to reduce the load on the fatigued musculature. The effects of fatigue on peripheral muscles include reduced maximal voluntary force production, velocity of muscle contraction, and power output [3,5-7].

Since the glenohumeral joint is minimally constrained by articular anatomy, shoulder muscles are largely responsible for the dynamic stability. When the shoulder muscles fatigue, joint mechanics become altered, thus possibly leading to rotator cuff pathologies [8]. Previous studies have shown that fatigue of the shoulder girdle musculature results in altered glenohumeral and scapulothoracic kinematics [1,4,9-12]. Others have reported decreased proprioceptive feedback with fatigue of the shoulder musculature [13]. Taken together, these data suggest that muscular fatigue impedes sensorimotor function and may predispose the shoulder to injury during activity [14].

To our knowledge, no standardized clinical endurance protocol has been developed to evaluate the effects of muscle fatigue on shoulder function. Such an endurance test could improve the examination and treatment for individuals with little or no pain during shoulder evaluation, but who complain of high level of pain or disabilities during work or sport/leisure activities. The evaluation of muscular performance in clinics is usually performed by measuring shoulder muscle strength using handheld or stationary dynamometers. However, there is not a one-to-one relationship between local muscular endurance and muscle strength, and endurance/fatigue tests are more reflective of functional use than isolated measure of muscle strength. There has been an abundance of research regarding muscle fatigue and how muscle fatigue can affect joint mechanics. However, no standardized endurance protocol has been proposed to evaluate, in clinics, the effects of fatigue. The purposes of this study were to establish a reliable protocol for objective assessment of shoulder muscle endurance and to examine the impact of low versus high standardization of methods on the reliability obtained.

## Methods

### Test Protocol Development

Assessment of local muscular endurance requires that an individual perform a series of repeated, submaximal contractions at a load that represents 50-80% of their maximum mean peak torque. Since there is no standardized clinical protocol for assessing shoulder muscular endurance, we reviewed the literature for approaches to maintain repeated muscles contractions. Magnitude of muscle fatigue is known to be associated with metabolic load which is a function of intensity of contraction, type of contraction (isometric, isotonic, concentric, eccentric), duty cycle (contraction: relaxation duration), muscle morphology and training status [15-17]. We considered each of these factors in pilot testing a variety of approaches to attain a protocol that a majority of normal healthy individuals would be able to perform, but that would assess fatigue. Thereafter, the final protocol was studied in two phases to assess the effects of standardization of the procedure itself.

### Subjects

Thirty-six healthy subjects volunteered to participate in the study. Subjects were all healthy young adults (mean age = 23.3 ± 2.8 years; range: 20 to 34 years) with no history of shoulder injuries to their dominant arm (see Table 1 for subjects characteristics). All the participants read and signed an informed consent form. This study was approved by the Hamilton Health Sciences Research Ethics Board.

**Table 1:** Baseline subject characteristics (mean ± standard deviation or n)

	All the subjects (n = 36)	
**Age (Years)**	23.3 ± 2.8	
**Gender (n)**	18 men/18 women	
**Dominant arm (n)**	27 right/9 left	
**Weight (kg)**	53.3 ± 12.1	

	**Low standardization** **(n = 15)**	**High Standardization** **(n = 21)**

**Age (Years)**	22.4 ± 1.4	24.1 ± 3.6
**Gender (n)**	6 men/9 women	12 men/9 women
**Dominant side (n)**	11 right/4 left	16 right/5 left
**Weight (kg)**	49.7 ± 13.4	56.6 ± 10.1

### Experimental Design

All subjects performed the tests on two separate occasions that were scheduled at least two days apart (mean = 2.4 ± 0.3 days, range: 2 to 4 days). At the first evaluation session all subjects performed baseline isokinetic and isometric shoulder strength assessment. All subjects performed five repetition maximum (5RM) concentric contractions to determine their 5RM isokinetic mean peak torque and three isometric maximal voluntary contractions (MVC). Then, they performed the shoulder endurance protocol. Immediately following performance of the shoulder endurance protocol, the decrement in 5RM isokinetic mean peak torque and isometric MVC were assessed. At the second evaluation session, each subject repeated the endurance protocol, followed by reassessment of their isokinetic and isometric shoulder strength. The evaluator was blinded to the data from the first session when retesting. All measures were performed on the dominant arm.

### Strength measurement

Isokinetic concentric mean peak torque of the shoulder internal and external rotators was measured using the Biodex System 3 dynamometer (Biodex Medical Systems, 20 Ramsay Road, Shirley, NY, 11967-47). The subjects stood next to the dynamometer, with their shoulder abducted 30° and their elbow flexed to 90°. The shoulder adapter and shoulder attachment, attached to the dynamometer, were used to secure the arm in this position. Before testing, each subject performed two practice trials on the Biodex. Then, each subject performed five maximal isokinetic repetitions of concentric internal/external rotation at 60°/sec. The mean peak torque values for the five repetitions were recorded for both internal and external rotation.

Maximal isometric strength of shoulder flexors and external rotators were tested using a hand-held dynamometer (HHD), the Lafayette Manual Muscle Test System (Lafayette Instrument Company, 3700 Sagamore Pkwy N. Lafayette, IN, 47904). Maximal isometric strength measurements were performed with the subject in a seated position, with both feet flat on the floor. Maximal isometric strength of the shoulder flexors was measured at 90°of shoulder flexion in the sagittal plane, with the elbow fully extended and the forearm in neutral position. Resistance was applied on the lateral aspect of the forearm just proximal to the styloid process. Maximal isometric strength of the shoulder external rotators was measured with the arm at the side and elbow flexed 90°. Resistance was applied on the dorsal aspect of the forearm just proximal to the head of the ulna. Three trials of isometric MVC were performed for each muscle group and the highest measure was recorded as 100% MVC.

### Endurance Protocol

The endurance protocol was performed on the Biodex with the subject in the same position as that used for the isokinetic strength measurements. The endurance protocol was performed in isotonic mode with the resistance set at 50% of each subject's 5RM mean peak torque as measured at baseline for each movement of shoulder external (ER) and internal rotation (IR). The resistances used during the second day of testing were identical to those established on Day 1. Each subject performed 60 continuous repetitions of IR/ER rotation. Subjects were asked to maintain the velocity during the protocol to at least 60°/sec and to perform maximal contractions throughout the endurance test (i.e. not to pace themselves). Subjects were given feedback on their velocity of movement. There was no maximal velocity for the test. Range of motion (ROM) was preset to the maximal internal and external rotation that each subject was comfortable using. The following criterion measures were extracted from the endurance protocol data: 1) the mean peak velocity in degrees/second, 2) the total work performed in joules (J), and 3) the decrement in work (i.e. fatigue) measured as the percentage difference in work capacity between the first third and the last third of the repetitions performed for the endurance protocol.

Fifteen of the 36 participants performed the study with lower standardization (low standardization subgroup); while the remaining 21 subjects performed the study with higher standardization (high standardization subgroup). In the lower standardization protocol, participants were instructed about the test position, number of repetitions (n = 60), and minimal speed to be maintained, as per protocol. In the higher standardization procedure, to reduce trunk movement during the endurance protocol, participants braced themselves with a strap to ensure proper posture and balance. In addition, ROM set on the Biodex Day 1 was also used on Day 2. Finally, consistent standardized verbal encouragement was provided throughout the testing to encourage subjects to give maximal effort throughout the endurance protocol. The experimenter instructed the subject to "try your best" at the start and again after each block of ten trials (i.e. at the beginning, and again after 10, 20, 30, 40, and 50 repetitions).

The modified 10-point version of the Borg rating of perceived exertion (RPE) [18] was used to measure self-reported feeling of exertion before and after the endurance protocol. Although this is a self-report measure, it has been shown to provide good estimate (r >0.86) of the actual heart rate during physical activity [19]. The scale ranged from 0 (no exertion at all) to 10 (maximal exertion). The Borg scale was administered verbally immediately before and after the endurance protocol on the first evaluation session.

### Statistical Analysis

Statistical analyses were performed for the whole group (n = 36), as well as the low-standardization (n = 15 subjects) and high-standardization (n = 21 subjects) subgroups. Paired *t*-tests were done to evaluate the effects of the endurance protocol by comparing maximal isometric and isokinetic strength before and after performing the fatiguing endurance protocol. The effect of the endurance protocol was also evaluated by examining the decline in total amount of work performed during the first third (first 20 trials of session one) of the endurance protocol compared to the total work during the last third of the protocol (last 20 trials of session one). Independent student *t*-tests were also used to compare the average peak velocity and decline in total work between low and high standardized subgroups. These two variables were selected for comparison between subgroups because they are less affected by gender than absolute strength measures.

The intersession reliability of the endurance protocol was determined by calculating reliability coefficients for criterion measures across sessions. Intraclass correlation coefficients (ICCs) Model (2,1) and their associated 95% confidence interval (CI) [20,21] were calculated. The absolute reliability was calculated with standard errors of measurement (SEM) and its 95%CI, and minimal detectable change (MDC) [22]. The MDC was calculated by multiplying the *z*-score corresponding to the level of significance, the square root of 2, and the SEM [23]. A *z*-score of 1.65 is used to achieve 90% confidence [23]. Confidence intervals that do not overlap indicate significant differences across compared subgroups. Test-retest reliability was also assessed using the Bland and Altman plotting method [24] and the corresponding bar charts [25]. This method plots individual differences in scores from test and retest against the mean difference of scores. The bar charts indicate the distribution of different sized retest differences. The limits of agreement (LOA) were estimated as the mean test-retest. All analyses were conducted with the SPSS software (Version 12; SPSS Inc, 233 S Wacker Dr, 11th Fl, Chicago, IL 60606). The alpha level was set at 0.05.

## Results

### Effect of the endurance protocol

Maximal isometric strength of the shoulder flexors and external rotators was significantly decreased after the endurance protocol for the whole group (*P *< 0.001), as well as for the low (*P *= 0.047) and high standardized subgroups (*P *< 0.001) (Tables 2 and 3). Conversely, isokinetic mean peak torque of the internal and external rotators remained unchanged. The total work performed during the last third of the protocol was significantly less than the first third of the protocol for the whole group (*P *< 0.001) as well as for the low (*P *= 0.041) and high (*P *= 0.037) standardized subgroups. The Borg RPE was increased following the endurance protocol (mean difference = 3.5/10; *P *< 0.001), reflecting increased feeling of perceived exertion. After the protocol, the subjects had a mean Borg RPE score of 8.3, which is associated with a "very hard" level of exertion. Average peak velocity (*P *< 0.001) and decline in total work (work fatigue, *P *< 0.02) during the endurance protocol were significantly lower in both muscle groups for low standardization when compared to high standardization protocols (Table 4).

**Table 2:** Effect of the endurance protocol on shoulder strength using a low-standardized method during session one (n = 15).

			Mean	SD	Percentage of change
**Isometric maximal strength (kg)**	**Flexion**	**Baseline**	22.7	5.0	
		**After protocol**	19.2*	3.8	- 15%
	**External Rotation**	**Baseline**	20.6	5.0	
		**After protocol**	18.8*	4.3	- 9%
**Isokinetic mean peak torque (Nm)**	**External Rotation**	**Baseline**	20.2	9.9	
		**After protocol**	21.5	10.4	+ 6%
	**Internal Rotation**	**Baseline**	23.7	10.3	
		**After protocol**	26.2	10.7	+ 11%
**Total work during endurance protocol (J)**	**External Rotation**	**First Third**	297.5	174.7	
		**Last Third**	215.2*	126.7	- 28%
	**Internal Rotation**	**First Third**	362.8	218.0	
		**Last Third**	267.6^¶^	126.6	- 26%

* Significant differences compared to the baseline strength measurements.

^¶ ^Significant differences compared to the total work in the first third of the endurance protocol.

**Table 3:** Effect of the endurance protocol on shoulder strength using a high-standardized method during session one (n = 21).

			Mean	SD	Percentage of change
**Isometric maximal strength (kg)**	**Flexion**	**Baseline**	20.2	8.1	
		**After protocol**	15.8*	6.2	- 22%
	**External Rotation**	**Baseline**	20.4	5.2	
		**After protocol**	16.9*	4.4	- 17%
**Isokinetic mean peak torque (Nm)**	**External Rotation**	**Baseline**	21.4	7.6	
		**After protocol**	21.3	7.8	0%
	**Internal Rotation**	**Baseline**	31.9	12.1	
		**After protocol**	31.4	12.0	-2%
**Total work during endurance protocol (J)**	**External Rotation**	**First Third**	395.7	189.1	
		**Last Third**	245.2*	86.4	- 38%
	**Internal Rotation**	**First Third**	592.8	299.6	
		**Last Third**	339.8^¶^	153.4	- 43%

* Significant differences compared to the baseline strength measurements.

^¶ ^Significant differences compared to the total work in the first third of the endurance protocol.

**Table 4:** Reliability of strength related measurements during the endurance protocol

Type of strength related measure	Muscular group	Group or subgroup	**Strength/fatigue**^**§**^	ICC	SEM	MDC90%
**Average peak velocity (°/sec)**	**External Rotation**	Whole group	142.3 ± 44.7	0.90	19.7	45.8
		Low-standardized	118.5 ± 42.7*	0.88	19.0	44.2
		High-standardized	158.1 ± 39.4*	0.85	18.9	44.0
	**Internal Rotation**	Whole group	161.7 ± 48.9	0.90	22.0	51.1
		Low-standardized	126.3 ± 41.7*	0.83	23.7	55.1
		High-standardized	185.3 ± 38.3*	0.82	19.9	46.3
**Total Work (J)**	**External Rotation**	Whole group	897.1 ± 411.9	0.96	111.6	259.5
		Low-standardized	781.0 ± 424.0	0.96	115.9	269.6
		High-standardized	974.5 ± 394.7	0.96	95.2	221.4
	**Internal Rotation**	Whole group	1230 ± 609	0.96	183.5	426.8
		Low-standardized	952.4 ± 495.3	0.94	179.1	416.5
		High-standardized	1415 ± 617	0.95	176.8	411.4
**Work Fatigue (%)**	**External Rotation**	Whole group	34.0 ± 18.9	0.83	11.5	26.8
		Low-standardized	22.5 ± 20.8*	0.84	13.3	30.9
		High-standardized	41.6 ± 13.2*	0.82	8.4	19.6
	**Internal Rotation**	Whole group	35.7 ± 21.7	0.78	13.2	30.8
		Low-standardized	25.7 ± 24.6*	0.78	14.7	34.1
		High-standardized	42.3 ± 17.1*	0.81	12.6	29.2

Abbreviations: ICC, intraclass correlation coefficient; SEM, standard errors of measurement; MDC, minimal detectable change. ^**§**^From session 1, Mean ± 1 standard deviation.

* Significant differences between low and high standardized subgroups.

### Reliability of the endurance protocol

**Figure 1:**
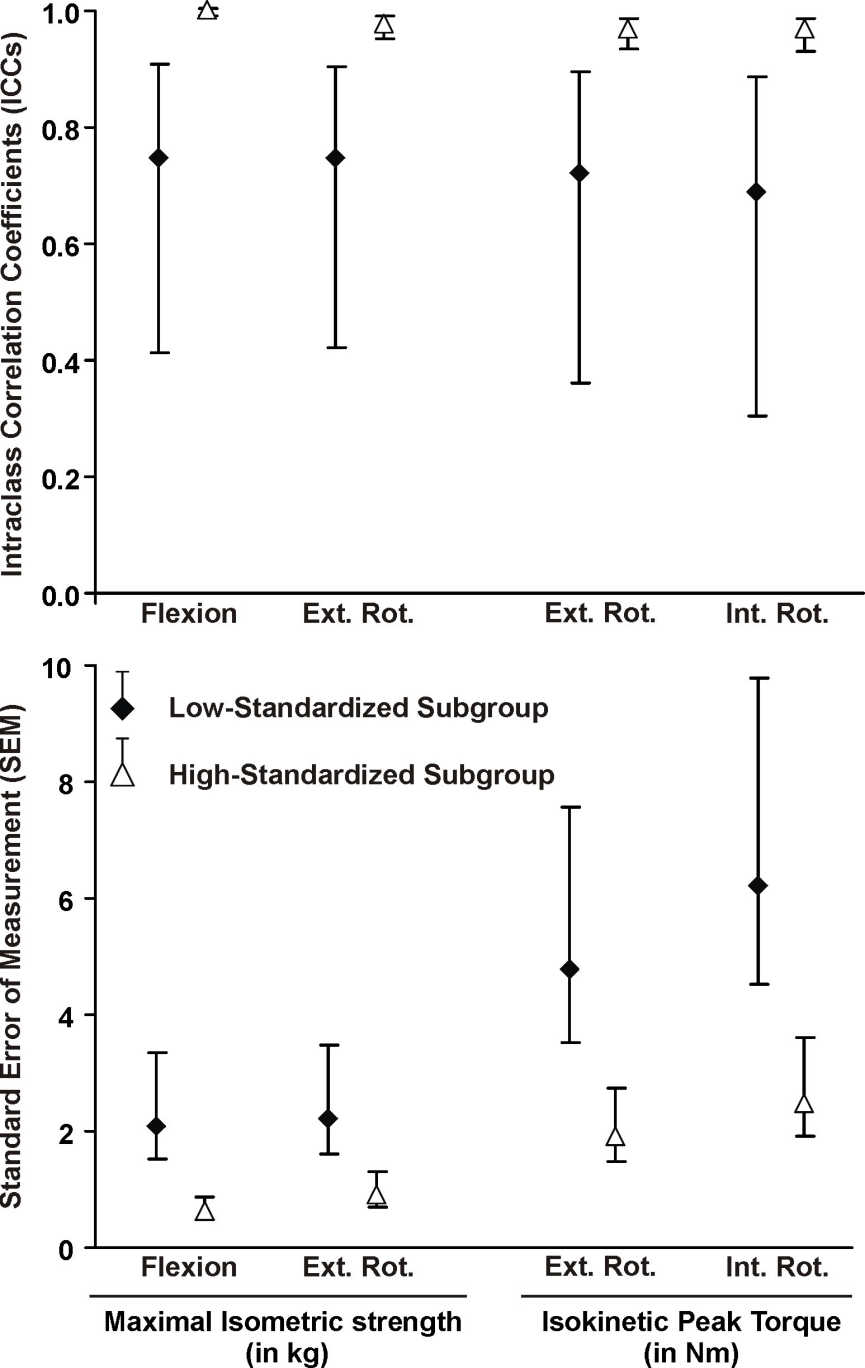
**Reliability of the strength measurements performed after the endurance protocol**. The ICCs, SEM and their 95% confidence interval are presented for the two subgroups: low-standardized subgroup (n = 15) and high-standardized subgroup (n = 21). Abbreviations: Ext. Rot, external rotation; Int. Rot.: internal rotation

**Figure 2:**
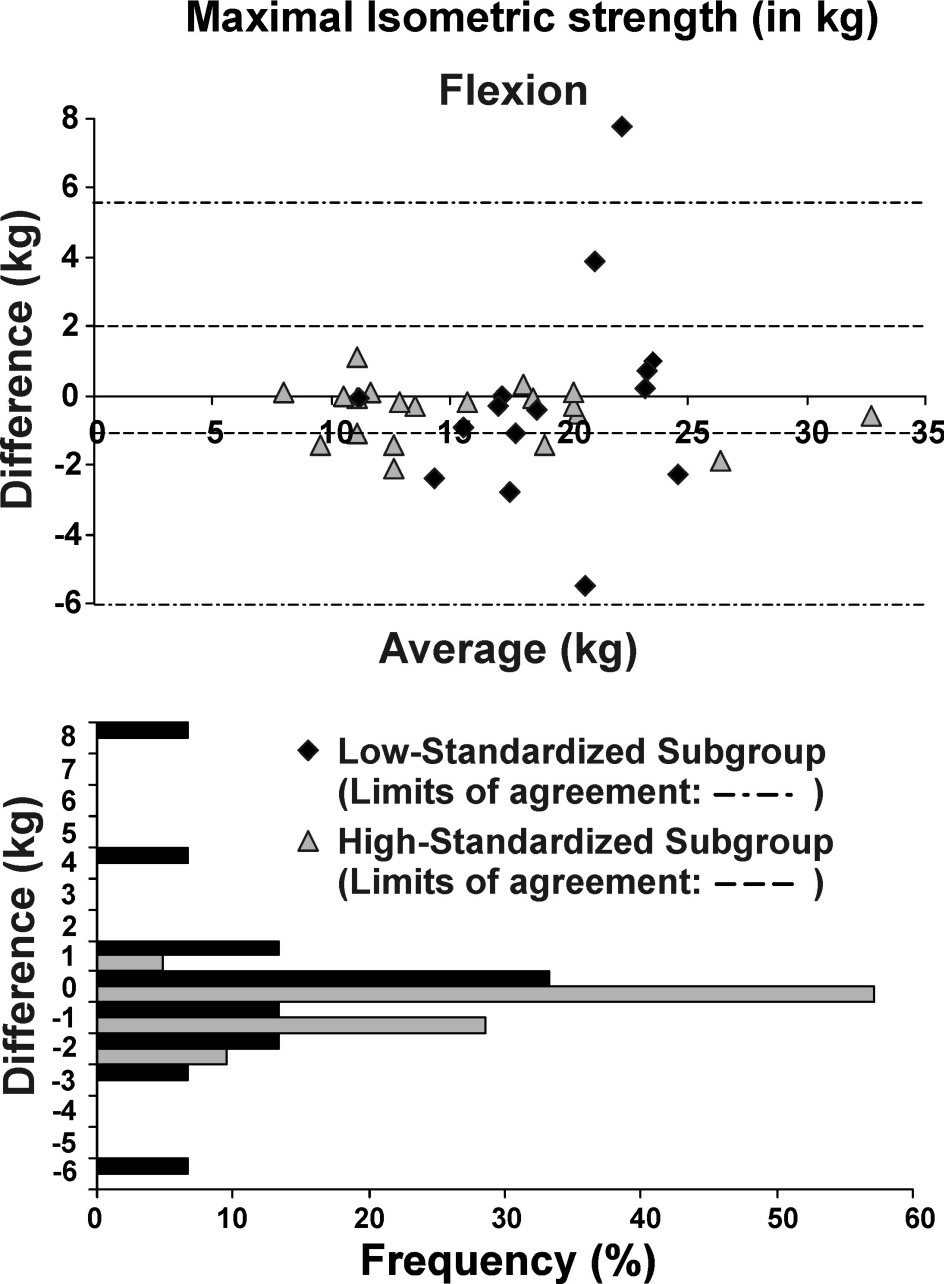
**Bland-Altman plots and corresponding bar charts for maximal isometric strength measurements in flexion performed after the endurance protocol**.

**Figure 3:**
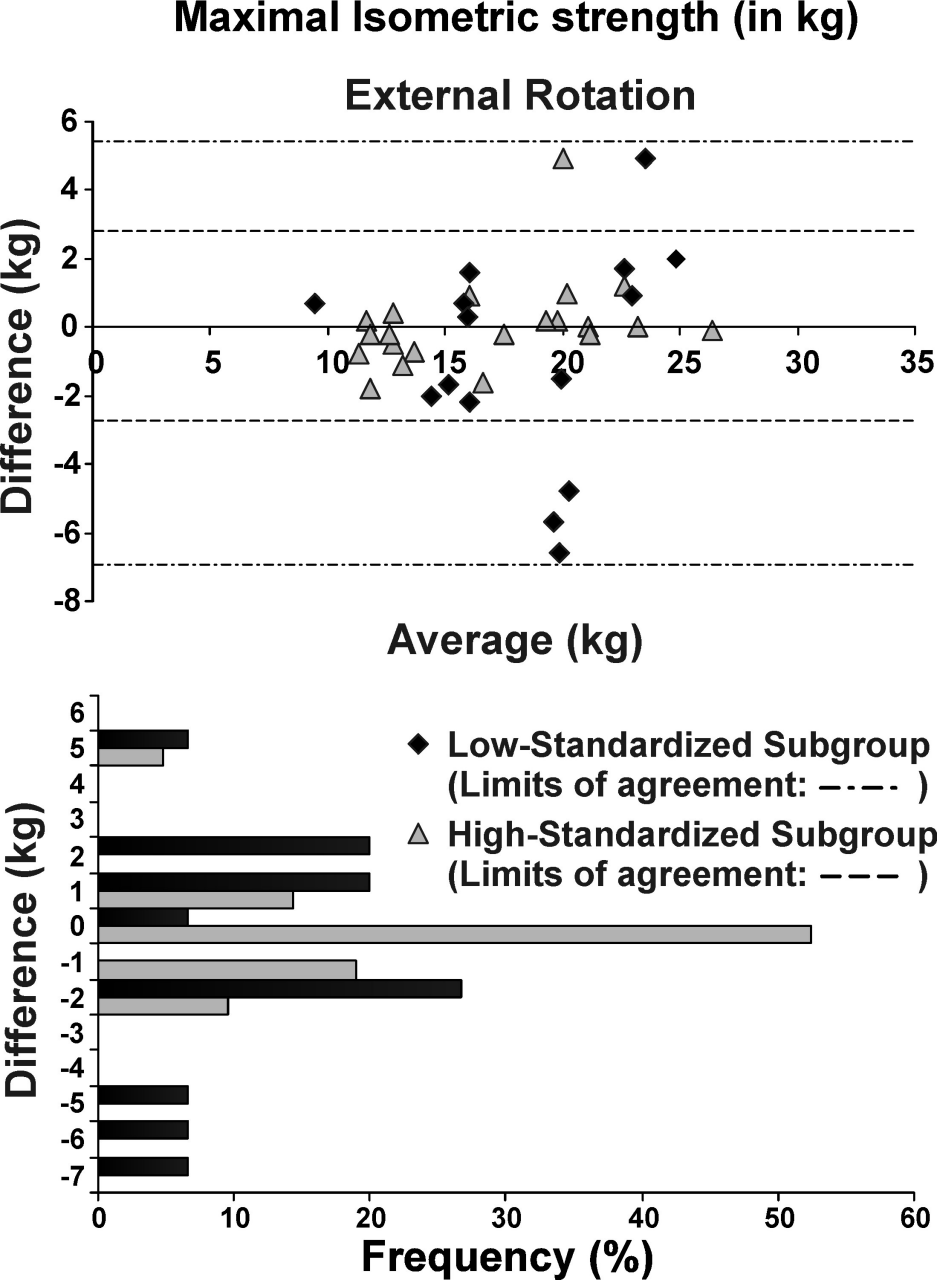
**Bland-Altman plots and corresponding bar charts for maximal isometric strength measurements in external rotation performed after the endurance protocol**.

**Figure 4:**
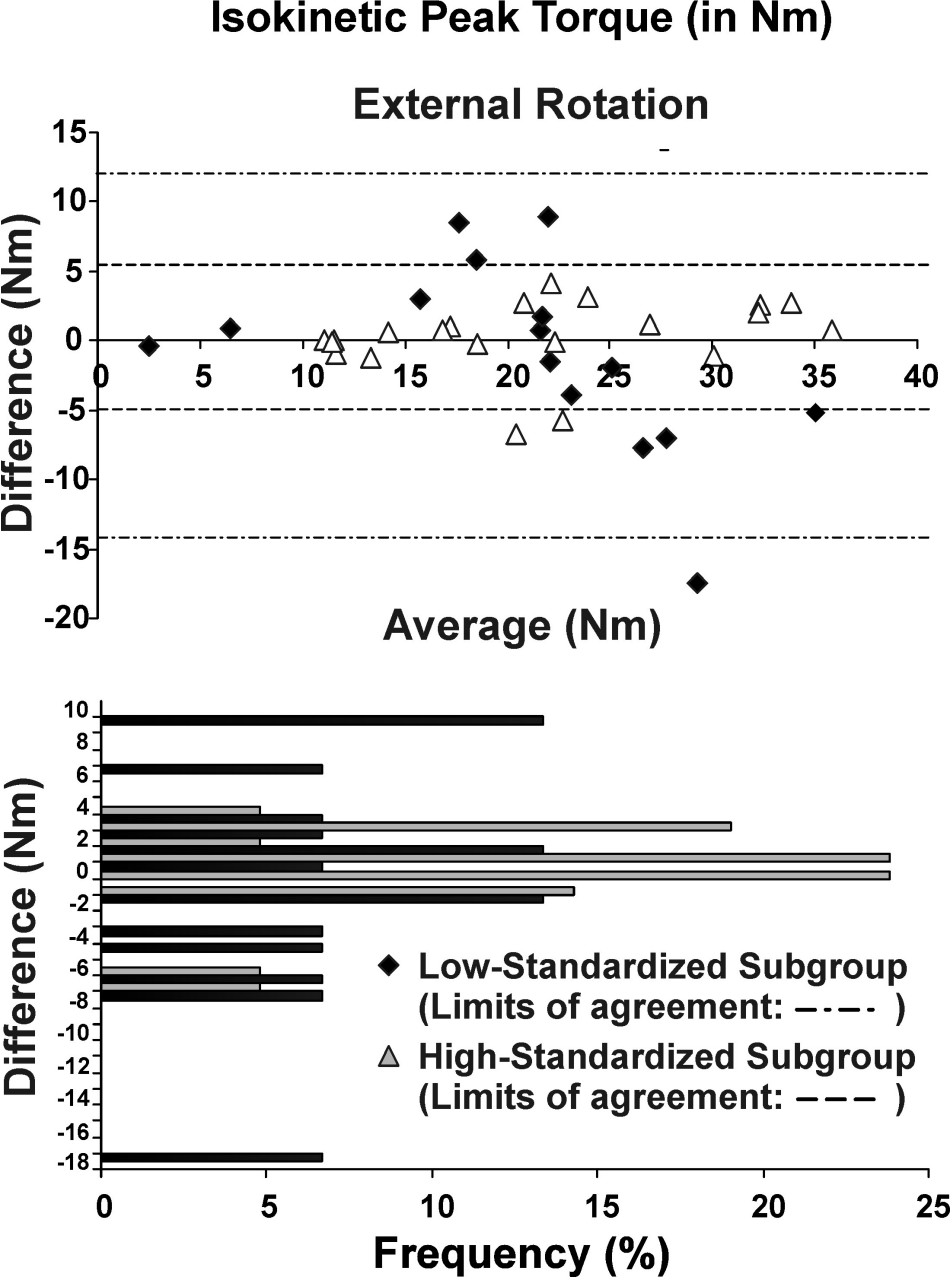
**Bland-Altman plots and corresponding bar charts for isotonic peak torque measurements in external rotation performed after the endurance protocol**.

**Figure 5:**
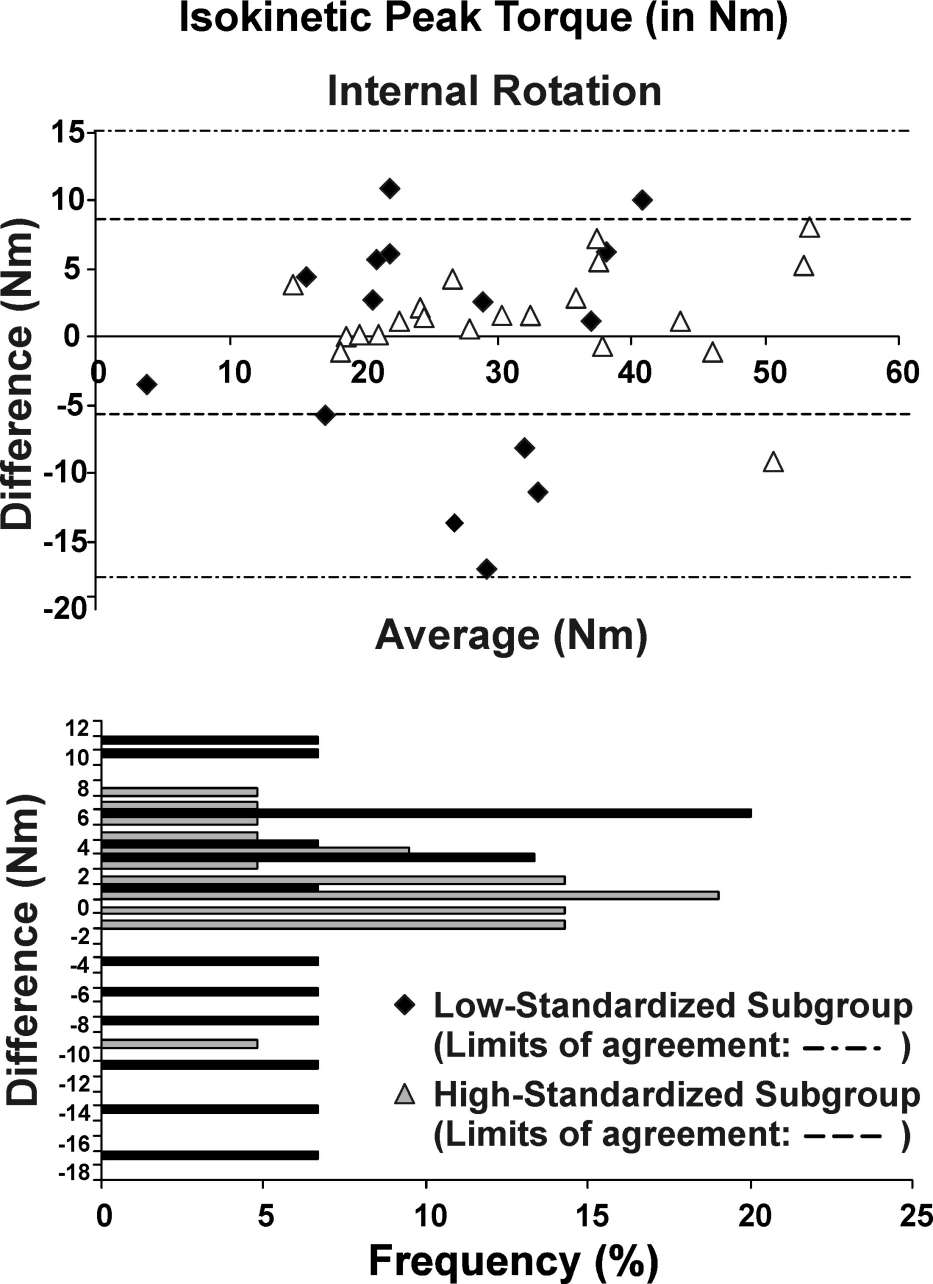
**Bland-Altman plots and corresponding bar charts for isotonic peak torque measurements in internal rotation performed after the endurance protocol**.

The test-retest reliability of the post-fatigue isokinetic and isometric strength measures was excellent (ICC >0.84) for the whole group and the high-standardized subgroup, but only moderate (ICC >0.69) for the low-standardized subgroup (Table 5). The SEM and MDC were lower (indicating better precision) for the high-standardized subgroup compared to low-standardized subgroup (Table 5). Overall absolute (SEM) and relative (ICCs) reliability was better with greater standardization (Figure 1). For the high-standardized subgroup, MDC represented 8-13% of the total isometric strength score; and 19-20% of the total mean peak torque (Table 5). The Bland and Altman plots revealed that test-retest differences were centered around zero regardless of the level of standardization (i.e. no bias indicated). However, the limits of the agreement were narrower (more precise) for the high-standardized subgroup (Figures 2, 3, 4 and 5).

**Table 5:** Reliability of the strength measurements performed after the endurance protocol

Type of strength measurement	Muscular group	Group or subgroup	**Strength**^**§**^	ICC	SEM	MDC90%
**Maximal Isometric strength (kg)**	**Flexion**	Whole group	17.2 ± 5.6	0.94^¶^	1.4^¶^	3.3
		Low-standardized	19.2 ± 3.8	0.75*	2.1*	5.0
		High-standardized	15.8 ± 6.2	0.99*^¶^	0.6*^¶^	1.3
	**External Rotation**	Whole group	17.7 ± 4.4	0.88^¶^	1.6	3.7
		Low-standardized	18.8 ± 4.3	0.75*	2.2*	5.2
		High-standardized	16.9 ± 4.4	0.98*^¶^	0.9*	2.2
**Isokinetic mean peak torque (Nm)**	**External Rotation**	Whole group	21.4 ± 8.8	0.84^¶^	3.4	7.9
		Low-standardized	21.5 ± 10.4	0.72*	4.8*	11.2
		High-standardized	21.3 ± 7.8	0.97*^¶^	1.9*	4.3
	**Internal Rotation**	Whole group	29.3 ± 11.6	0.86^¶^	4.4	10.3
		Low-standardized	26.2 ± 10.7	0.69*	6.2*	14.4
		High-standardized	31.4 ± 12.0	0.97*^¶^	2.5*	5.9

Abbreviations: ICC, intraclass correlation coefficient; SEM, standard errors of measurement; MDC, minimal detectable change.

^**§**^From session 1, Mean ± 1 standard deviation

* Significant differences between the standardized and the non-standardized subgroups.

^¶ ^Significant differences between the whole group and the standardized subgroups.

For maximal isometric strength in flexion and external rotation, 100% and 95% of the subjects, respectively, had test-retest differences of less than +/- 2 kg in the high-standardized subgroup, while 73% and 74%, respectively, had test-retest difference of less than +/- 2 kg in the low-standardized subgroup (Figures 2 and 3). As for isokinetic mean peak torque in external and internal rotation, 67% and 62% of the subjects respectively, had test-retest difference of less than +/- 2 N·m in the high-standardized subgroup, while only 40% and 7%, respectively, had test-retest differences of less than +/- 2 N·m in the low-standardized subgroup (Figures 4 and 5).

**Figure 6:**
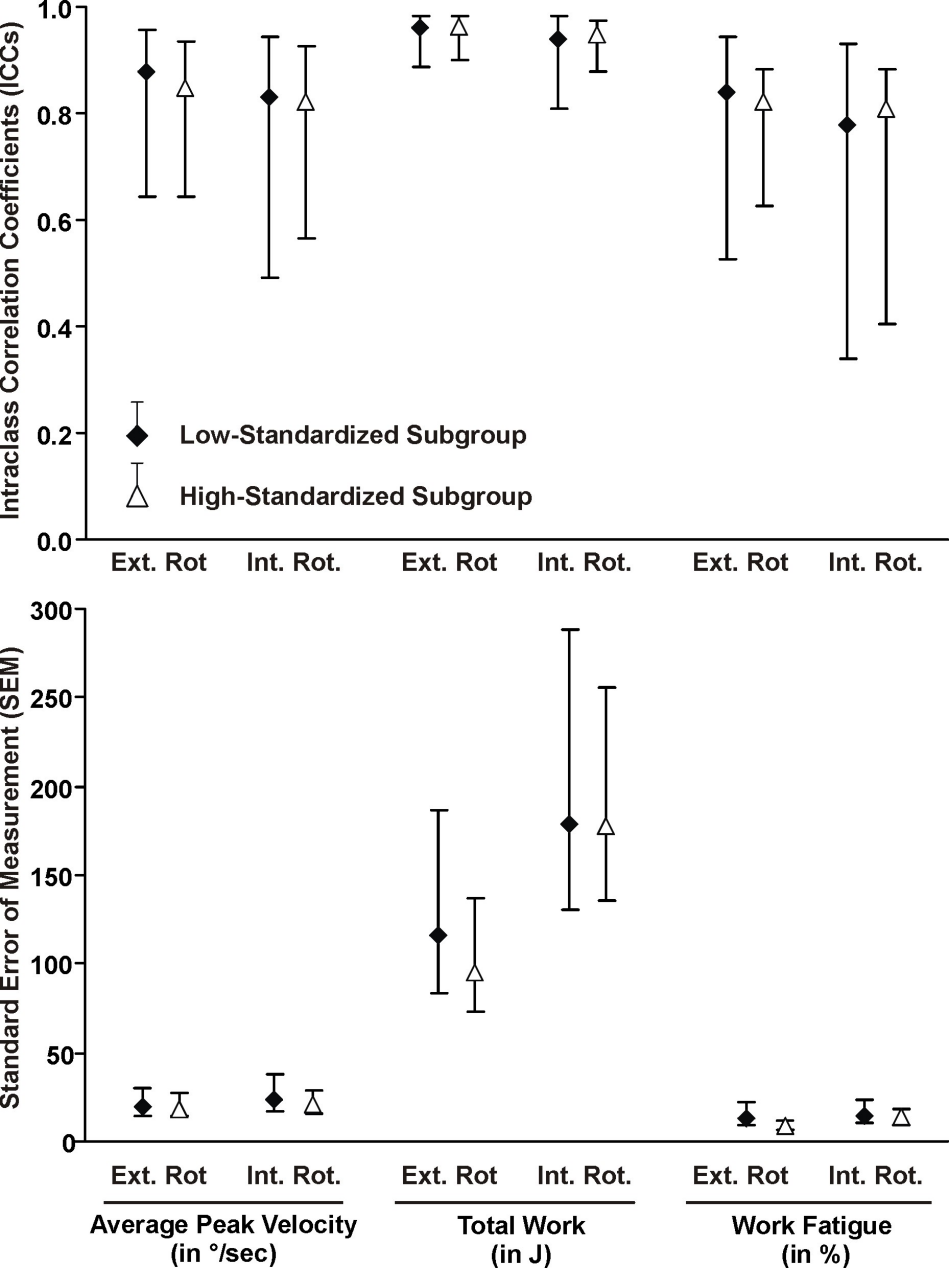
**Reliability of the strength related measures during the endurance protocol**. The ICCs, SEM and their 95% confidence interval are presented for the two subgroups: low-standardized subgroup (n = 15) and high-standardized subgroup (n = 21). Abbreviations: Ext. Rot, external rotation; Int. Rot.: internal rotation

The muscular endurance protocol measures demonstrated excellent reliability (ICC >0.80) for isokinetic measurement of mean peak velocity and total work, and good to excellent reliability (ICC >0.78) for work fatigue (Table 4 and Figure 6).

## Discussion

This study established that a newly proposed isotonic protocol to evaluate local muscular endurance of the shoulder can be consistently performed by healthy individuals across a test-retest period as the resultant effects of fatigue were reproducible over time. Greater precision and reliability coefficients can be obtained by using a more standardized and stabilized test protocol. The protocol was performed without difficulty by all volunteers and took less than 10 minutes to perform, suggesting that it might be feasible for clinical practice. This protocol could be used to induce local muscular fatigue in order to evaluate the effects of fatigue on shoulder kinematics or to evaluate changes in shoulder muscle endurance following rehabilitation.

Other endurance protocols have been used to evaluate the effects of shoulder muscle fatigue. For example, Tsai et al. [11] assessed fatigue during external rotation using Thera-band. In their protocol, repeated muscle contractions were performed using a single resistance level at a rate of approximately 1 Hertz until the subjects could no longer perform the task and demonstrated a minimum of 25% reduction in isometric torque production. A disadvantage of this protocol is the lack of standardization inherent in the use of Thera-band resistance and its mismatch to the length tension relationship of the muscle. Ebaugh et al. [1] reported a more functional fatigue protocol that consisted of three specific tasks: 1) with their arms elevated to 45°, the subjects manipulated small objects for 2 minutes; 2), subjects raised and lowered their tested arm against a resistance for 20 repetitions; 3) subjects raised and lowered their arm through a diagonal pattern against resistance for 20 repetitions. The subjects rotated through the three activities until they were unable to continue or failed to correctly perform the tasks. The average length of time that subjects performed the fatigue protocol was 10 min and 44 s and fatigue was observed with a reduction of 8% in the median power frequency. The length of test would suggest that the physiological system evaluated was aerobic in nature and not local muscular endurance. Szucs et al. [12] used a task to fatigue the serratus anterior by holding a push-up plus position. Subjects held this position until they stopped due to fatigue. These protocols were developed for specific research studies that were designed to evaluate the effects of fatigue on shoulder kinematics, and not for clinical purposes. To our knowledge, their reliability has not been evaluated.

The changes in muscular performance observed during and after our local muscular endurance protocol suggests that the protocol did result in fatigue of the scapulohumeral muscles. First, the total work performed during the last third of the protocol was 42% lower than during first third of the protocol. Another sign of fatigue was the decrease of more than 17% that occurred in maximal isometric strength (MVC) pre-post test. Subjects self-reported exertion scores indicated an exertion level that was "very hard". Therefore, the proposed endurance protocol not only resulted in shoulder fatigue, but it caused subjects to perceive an increase in self-reported exertion. Together, these findings support the validity of our construct that the protocol should physiologically produce local muscular fatigue. This is one of the few protocols available that uses a relative load to tax each individual based on their maximum strength (i.e. 50% of isokinetic 5 RM) and thus can be used with a range of individuals with shoulder disabilities.

However, one observation did not indicate muscle fatigue: minimal changes were observed in the isokinetic mean peak torque after the endurance protocol. It is impossible to be confident about why this phenomenon occurred, but we postulate that three factors may have contributed. First, our subjects were not familiar with the test protocol or Biodex and thus may not have been able to produce a true maximal contraction on their first peak torque assessment. Familiarity obtained with the device during the testing procedure might have helped them to perform better (despite muscle fatigue) when the peak torque was reassessed. A second issue was the time gap between completion of the endurance test and our ability to assess isokinetic mean peak force (one minute set-up time). If our fatigue protocol produces short-term fatigue then some muscle recovery may have occurred during this time interval. We feel the latter is less likely to be true given that isometric strength scores obtained after the isokinetic testing were able to demonstrate fatigue. Finally, subjects may have paced themselves throughout the test in order to be able to complete the entire 60 repetitions. This would suggest that learning or pacing may be important contributors. However, we would suggest that when assessing local muscular endurance, the measures of work and maximal isometric strength are more important criterion measures. Thus for isokinetic dynamometers, it appears that the use of total work (a measure produced by the manufacturer's software) is a better measure of fatigue.

Although standardization is accepted as an important component of enhancing reliability and validity, few studies have addressed its impact. Furthermore, it is unclear what level of standardization is needed. This study highlights quantitatively the impact of greater levels of standardization and stabilization. Standardization provided higher strength scores and enhanced reliability coefficients. Higher standardization was achieved with three simple modifications to the protocol: participants held on to a strap to ensure proper posture and balance, the same ROM for the endurance protocol was used on the test and retest, and consistent verbal encouragement were provide throughout the testing. The 25% increase in average peak velocity highlights the importance of verbal encouragement during testing. A standardized protocol and sustained effort also led to 18% more fatigue during the test and 8% greater reduction in post-test maximal strength. Previous studies have also shown that frequent verbal encouragement leads to significantly greater maximum effort than when no encouragement is given or when the encouragement is infrequent [26].

Lack of standardization is thought to contribute to random error, making it more difficult to find true differences between groups in research studies, or assess changes in individual patients over time. Our data would confirm this notion, since no substantial bias was detected by the Bland-Altman plots during testing. However, greater precision and higher ICCs were obtained with greater standardization. This study also confirms that the Biodex 3 is reliable for isotonic shoulder testing of both strength and local muscle endurance. Others have reported reliability for strength scores using a stationary dynamometer [27-29].

The MDC is important to consider when evaluating change in a patient's status since it can be used to determine whether the change is clinically meaningful [30]. For example, if the same patient who had an external rotation maximal isometric strength of 19 kg following the endurance protocol on the initial evaluation has a maximal isometric strength of 24 kg during reassessment 6 weeks later, the clinician will be able to state confidently that the patient has demonstrated statistically meaningful improvement because the change of 5 kg is greater than the MDC value (2.2 kg).

This study was developmental and has inherent limitations. First, only healthy subjects were evaluated. This is a first critical step in assessing test performance and, given that local muscle endurance was our construct of interest, we were able to observe a variety of performance even within uninjured individuals. However, future studies should also look at how the test is performed by patients with shoulder pathology. We determined the test was valid based on muscle performance, but future studies might provide more direct validation using electromyographic analysis [8]. Finally, the possibilities of combination for the endurance protocol parameters (relative load as a % of 5RM or MVC, number of repetitions, maximal/minimal speed or resistance, the duty cycle) were infinite. We used physiological rationale from human and animal studies of muscle fatigue and pilot testing to establish the most feasible approach that would produce fatigue and be reasonable for use in clinical practice. Despite our promising results, we cannot be confident that the test parameters do not require further optimization. Although we recognize that other combinations are potentially viable options for assessing endurance, we believe that establishing a reliable protocol that can be used by others has value.

## Conclusions

An endurance protocol that requires patients to perform 60 repetitions of isotonic contraction at 50% of their maximal isokinetic mean peak torque was found to produce muscular fatigue as indicated by decrements in mean peak torque and muscle work in healthy individuals following performance of the fatiguing protocol. The protocol was reliable and had acceptable precision. Greater fatigue and better reliability were achieved with higher levels of protocol standardization. Future studies should focus on evaluating the feasibility of using this protocol to evaluate individuals with various shoulder pathologies.

## List of abbreviations

CI: Confidence interval; ER: External rotation; HHD: Hand-held dynamometer; ICC: Intraclass correlation coefficient; IR: Internal rotation; LOA: limits of agreement; MDC: Minimal detectable change; MVC: Maximal voluntary contraction; ROM: Range of motion; RPE: Rating of perceived exertion; SEM: Standard error of measurement; 5RM: five repetitions maximum

## Competing interests

The authors declare that they have no competing interests.

## Authors' contributions

JSR: participated in the design of the study, the analysis and the interpretation of data and drafted the manuscript. BM: participated in the design of the study, carried out the acquisition and drafted the manuscript. JCM: participated in the design of the study, the analysis and the interpretation of data and drafted the manuscript. LJW: participated in the design of the study, the analysis and the interpretation of data and drafted the manuscript.

All authors read and approved the final manuscript.
